# Comparative Effectiveness of Sodium-Glucose Co-transporter 2 Inhibitors and Thiazolidinediones in Reducing Adverse Cardiovascular Events in Type 2 Diabetes

**DOI:** 10.7759/cureus.93303

**Published:** 2025-09-26

**Authors:** Arshad Ali, Uzair Sohail, Talha Rao, Faryal Tariq, Abdul Moeed Baig, Syeda Ambreen Fatima

**Affiliations:** 1 Internal Medicine, Hayatabad Medical Complex, Peshawar, PAK; 2 Medicine, HITEC Institute of Medical Sciences, Taxila, PAK; 3 Medicine, Tehsil Headquarter Hospital, Mailsi, PAK; 4 Medicine, East Kent Hospital University Foundation Trust, England, GBR; 5 Medicine, Gujranwala Medical College, Gujranwala, PAK; 6 Medicine, Hywel Dda University Health Board, Withybush General Hospital, Pembrokeshire, GBR

**Keywords:** cardiovascular events, comparative effectiveness, sglt-2 inhibitors, thiazolidinediones, type 2 diabetes

## Abstract

Background

Cardiovascular disease is the leading cause of morbidity and mortality among patients with type 2 diabetes mellitus (T2DM). While both sodium-glucose co-transporter 2 inhibitors (SGLT-2is) and thiazolidinediones (TZDs) have demonstrated cardiovascular effects, comparative data in real-world South Asian populations remain limited.

Objective

This study aimed to compare the effectiveness of SGLT-2is and TZDs in reducing adverse cardiovascular events (ACVEs) in patients with T2DM.

Methodology

A prospective, comparative cohort study was conducted at HITEC Institute of Medical Sciences, Taxila, from January to December 2024. A total of 240 T2DM patients with high cardiovascular risk were recruited and divided equally into two groups: one receiving SGLT-2is (dapagliflozin or empagliflozin) and the other receiving TZDs (pioglitazone). Baseline clinical and biochemical parameters were recorded and followed for 12 months. Outcomes included changes in blood pressure, body mass index (BMI), glycemic and lipid profiles, renal function, and incidence of major adverse cardiovascular events (MACE).

Results

At baseline, both groups were comparable in age (mean 56.4±9.8 years), sex distribution (58% male), and hypertension duration (8.6±5.1 years). After 12 months, the SGLT-2is group showed greater reductions in systolic blood pressure (130.5±9.1 mmHg vs. 134.7±10.2 mmHg; p<0.05), BMI (28.7±3.4 vs. 29.9±3.6 kg/m²), and HbA1c (7.1±0.7% vs. 7.4±0.8%). Lipid profiles and fasting glucose also improved more in the SGLT-2is group. The incidence of MACE was significantly lower in the SGLT-2is group (8.3%) compared to the TZD group (21.7%), with fewer cardiovascular deaths, myocardial infarctions, strokes, and heart failure hospitalizations.

Conclusion

SGLT-2is demonstrated superior cardiometabolic outcomes and a lower risk of ACVEs compared to TZDs, supporting their preferential use in patients with T2DM at elevated cardiovascular risk.

## Introduction

Cardiovascular disease (CVD) remains the leading cause of morbidity and mortality among patients with type 2 diabetes mellitus (T2DM) worldwide, accounting for a significant proportion of healthcare burden [1]. Among Asian populations, including Pakistan, the burden of cerebrovascular events, particularly stroke, is disproportionately higher compared to Western populations, where coronary artery disease predominates [2]. Because of these regional differences, comparative effectiveness data for sodium-glucose co-transporter 2 inhibitors (SGLT-2is) and thiazolidinediones (TZDs) are particularly needed in South Asia.

SGLT-2is have emerged as a novel class of antidiabetic drugs with significant cardiovascular benefits. They act by inhibiting renal glucose reabsorption in the proximal tubules, leading to glucosuria, natriuresis, and subsequent improvements in glycemic control, blood pressure, and cardiovascular outcomes. Large randomized controlled trials (RCTs) such as EMPA-REG OUTCOME and CANVAS have demonstrated that SGLT-2is reduce the risk of major adverse cardiovascular events (MACE), especially cardiovascular death and heart failure (HF) hospitalization [3]. Early meta-analyses suggested a possible increase in stroke risk, but subsequent studies did not confirm this, and the effect is now considered neutral [4,5].

TZDs, particularly pioglitazone, act as peroxisome proliferator-activated receptor gamma (PPARγ) agonists and have demonstrated efficacy in improving insulin sensitivity and exerting anti-atherosclerotic effects [6]. Clinical evidence, including the PROactive and IRIS trials, indicates that pioglitazone reduces recurrent stroke and composite cardiovascular outcomes, especially in patients with prior cerebrovascular disease [7]. However, its cardiovascular safety profile, particularly in patients without previous stroke, has been more nuanced and warrants further exploration.

Despite the known cardioprotective properties of both drug classes, direct comparisons between SGLT-2is and TZDs in real-world settings are scarce. In contrast, real-world observational data, such as a large Korean nationwide study, revealed no significant difference in stroke or myocardial infarction (MI) rates between the two groups, although SGLT-2is significantly reduced HF hospitalizations [8]. Nonetheless, heterogeneity in study populations, baseline cardiovascular risk, drug dosing, and ethnic variability underscores the need for region-specific prospective evaluations. Given the paucity of head-to-head prospective studies directly comparing SGLT-2is and TZDs, especially in South Asian populations, this study aims to be among the first to prospectively evaluate their comparative effectiveness in reducing adverse cardiovascular events (ACVEs) in patients with T2DM at a tertiary care center in Pakistan, thereby adding unique value to the existing literature.

## Materials and methods

This prospective, comparative cohort study was conducted at HITEC Institute of Medical Sciences, Taxila, between January 2024 and December 2024, to evaluate and compare the effectiveness of SGLT-2is and TZDs in reducing ACVEs in patients with T2DM. The study adhered to ethical standards and was approved by the Institutional Review Board of HITEC Institute of Medical Sciences, Taxila (Approval Ref: 2023/957, dated 12-12-2023). Written informed consent was obtained from all participants prior to enrollment. 

The sample size was calculated using OpenEpi software (www.OpenEpi.com), based on the expected incidence reduction of MACE from prior studies comparing SGLT-2is and TZDs. Assuming a power of 80%, a confidence level of 95%, and an effect size of 15% difference in ACVEs, the calculated sample size was 240 patients, with 120 patients in each treatment arm. An additional 10% was added to account for potential dropouts, resulting in a final target sample of 264 patients. During follow-up, 24 patients were excluded due to loss to follow-up (n=12), withdrawal of consent (n=7), and protocol violations (n=5). Therefore, the final analysis was conducted on 240 patients.

Patients were recruited using a non-probability consecutive sampling method from the endocrinology and cardiology outpatient departments. Patients aged 40 to 70 years, diagnosed with T2DM for at least one year, with established CVD or at high cardiovascular risk (as defined by the American Diabetes Association criteria), and who had not previously been treated with either SGLT-2is or TZDs were eligible for inclusion. Patients were excluded if they had type 1 diabetes mellitus, end-stage renal disease (eGFR <30 mL/min/1.73 m²), active malignancy, hepatic dysfunction, HF with reduced ejection fraction (for TZD group), pregnancy, or were unable to provide informed consent.

Following recruitment, patients were allocated into two groups based on their prescribed treatment regimen: Group A received SGLT-2is (dapagliflozin 10 mg or empagliflozin 10 mg once daily), and Group B received TZDs (pioglitazone 15-30 mg once daily), in addition to standard antidiabetic therapy as required to achieve glycemic targets. This was a non-randomized allocation based on clinical prescription practices; patients and treating physicians were not blinded. To minimize potential selection bias and confounding, multivariable Cox regression models were applied to adjust for baseline demographic and clinical covariates.

Baseline data collection included demographic details, diabetes duration, comorbidities, smoking status, body mass index (BMI), blood pressure, and lipid profile. Laboratory investigations were performed using standardized techniques. HbA1c was measured using high-performance liquid chromatography (HPLC), while fasting blood glucose (FBG) and lipid profile (total cholesterol, low-density lipoprotein (LDL), high-density lipoprotein (HDL), and triglycerides) were analyzed by enzymatic colorimetric methods. Renal function was assessed through serum creatinine and eGFR calculated using the CKD-EPI formula. Baseline concomitant cardiovascular medications (including statins, ACE inhibitors/ARBs, antiplatelet agents, beta-blockers, and GLP-1 receptor agonists) were recorded, and their distribution between treatment groups is provided in the baseline characteristics table.

Electrocardiograms (ECGs) and echocardiograms were performed at baseline and repeated if clinically indicated. MACE were defined as a composite of cardiovascular death, non-fatal MI, non-fatal stroke, and hospitalization for HF. Event adjudication was performed by an independent cardiologist who was blinded to treatment assignment, patient identity, and study hypothesis.

Statistical analysis was conducted using SPSS version 26.0 (IBM SPSS Statistics for Windows, IBM Corp., Armonk, NY). Continuous variables were expressed as mean±SD and compared using Student’s t-test or Mann-Whitney U test, depending on data normality. Categorical variables were presented as frequencies and percentages and compared using chi-square or Fisher’s exact test. Time-to-event data for MACE were analyzed using Kaplan-Meier survival curves and compared with the log-rank test. Cox proportional hazards regression was used to adjust for potential confounders such as age, sex, BMI, baseline HbA1c, blood pressure, lipid levels, and concomitant medications. A p-value of <0.05 was considered statistically significant.

All procedures and analyses were performed in accordance with the ethical principles of the Declaration of Helsinki. Data confidentiality was maintained throughout the study, and only de-identified data were used for analysis.

## Results

A total of 240 patients were enrolled in the study and equally divided into two treatment arms: 120 patients received SGLT-2is (Group A), and 120 patients received TZDs (Group B). Baseline demographic and clinical characteristics of both groups were comparable, with no statistically significant differences except for smoking status. The mean age was 57.5±7.0 years in the SGLT-2is group and 58.5±8.0 years in the TZD group. Males comprised 56 (47%) patients in each group. The mean BMI was slightly lower in the SGLT-2is group (29.7±3.5 kg/m²) compared to the TZD group (30.9±3.8 kg/m²). The mean duration of T2DM was 8.0±2.9 years in the SGLT-2is group versus 8.7±3.1 years in the TZD group. Smoking prevalence was significantly higher in the TZD group, 43 (36%), compared to 23 (19%) in the SGLT-2is group (p=0.005). Mean baseline systolic blood pressure (SBP) was 135.9±11.2 mmHg in the SGLT-2is group and 138.3±12.0 mmHg in the TZD group, while diastolic blood pressure (DBP) was 84.4±6.8 mmHg and 85.2±7.1 mmHg, respectively (Table 1).

**Table 1:** Baseline characteristics of study participants ^*^P<0.05 is considered statistically significant. Values are expressed as mean±SD for continuous variables and frequency (%) for categorical variables. An independent samples t-test was used for continuous variables; a Chi-square test was applied for categorical variables. SGLT-2is: sodium-glucose cotransporter-2 inhibitors; TZDs: thiazolidinediones; BMI: body mass index; T2DM: type 2 diabetes mellitus; SBP, systolic blood pressure; DBP, diastolic blood pressure

Parameter	SGLT-2is (n=120)	TZDs (n=120)	Test statistic	P-value
Age (years, mean±SD)	57.5±7.0	58.5±8.0	t=-0.82	0.412
Male (n, %)	56 (47%)	56 (47%)	χ²=0.00	1
BMI (Kg/m², mean±SD)	29.7±3.5	30.9±3.8	t=-1.96	0.052
Duration of T2DM (years, mean±SD)	8.0±2.9	8.7±3.1	t=-1.38	0.168
Smokers (n, %)	23 (19%)	43 (36%)	χ²=7.86	0.005^*^
SBP (mmHg, mean±SD)	135.9±11.2	138.3±12.0	t=-1.45	0.148
DBP (mmHg, mean±SD)	84.4±6.8	85.2±7.1	t=-0.78	0.439

After 12 months of therapy, the SGLT-2i group demonstrated statistically significant improvements in clinical parameters compared to the TZD group. Mean SBP was reduced to 130.5±9.1 mmHg in the SGLT-2is group compared to 134.7±10.2 mmHg in the TZD group. DBP followed a similar trend. BMI reduced more in the SGLT-2is group (28.7±3.4 kg/m²) than in the TZD group (29.9±3.6 kg/m²), although not reaching statistical significance (Table 2).

**Table 2:** Clinical outcomes at 12 months *p<0.05 is considered statistically significant. Values are presented as mean±SD for the SGLT-2i group (n=120) and the TZD group (n=120). Between-group comparisons were performed using two-tailed independent-samples Student’s t-tests. TZD, thiazolidinedione; SGLT-2is, sodium-glucose co-transporter 2 inhibitors; SBP, systolic blood pressure; DBP, diastolic blood pressure; BMI, body mass index

Parameter	SGLT-2is (n=120)	TZDs (n=120)	t-value	p-value
SBP (mmHg)	130.5±9.1	134.7±10.2	2.32	0.021*
DBP (mmHg)	82.2±5.8	83.5±6.1	1.62	0.106
BMI (kg/m²)	28.7±3.4	29.9±3.6	1.79	0.074

Biochemically, patients on SGLT-2is had significantly better glycemic control. Mean HbA1c was 7.1±0.7% in the SGLT-2is group compared to 7.4±0.8% in the TZD group (p<0.05). FBG levels were lower in the SGLT-2is group (115.6±18.4 mg/dL) than the TZD group (123.8±19.7 mg/dL). Favorable lipid changes were observed in the SGLT-2is group, with lower total cholesterol (178.2±30.1 vs. 190.3±32.7 mg/dL) and LDL levels (98.6±24.8 vs. 107.3±27.1 mg/dL). HDL was slightly higher in the SGLT-2is group (48.2±10.1 mg/dL vs. 46.5±9.7 mg/dL). Renal function remained stable in both groups with comparable serum creatinine and eGFR values (Table 3).

**Table 3:** Biochemical parameters at 12 months *p<0.05 is considered statistically significant. Values are presented as mean±SD for the SGLT-2i group (n=120) and the TZD group (n=120). Between-group comparisons used two-tailed independent-samples Student’s t-tests. TZD, thiazolidinedione; SGLT-2is, sodium-glucose co-transporter 2 inhibitors; LDL, low-density lipoprotein; HDL, high-density lipoprotein; eGFR, estimated glomerular filtration rate

Parameter	SGLT-2is (n=120)	TZDs (n=120)	t-value	p-value
HbA1c (%)	7.1±0.7	7.4±0.8	t=2.10	0.038*
Fasting glucose (mg/dL)	115.6±18.4	123.8±19.7	t=2.96	0.004*
Total cholesterol (mg/dL)	178.2±30.1	190.3±32.7	t=2.67	0.008*
LDL (mg/dL)	98.6±24.8	107.3±27.1	t=2.32	0.021*
HDL (mg/dL)	48.2±10.1	46.5±9.7	t=1.12	0.262
Triglycerides (mg/dL)	155.7±45.3	163.4±47.2	t=1.28	0.202
Creatinine (mg/dL)	0.91±0.12	0.93±0.14	t=1.10	0.272
eGFR (mL/min/1.73m²)	84.3±9.6	82.7±10.3	t=1.12	0.264

Cardiovascular outcomes during the follow-up period showed a clear advantage in favor of the SGLT-2i group. The incidence of MACE, defined as a composite of cardiovascular death, non-fatal MI, non-fatal stroke, and hospitalization for HF, was significantly lower in the SGLT-2is group (8.3%) compared to the TZD group (21.7%). Specifically, cardiovascular deaths occurred in 2 (1.7%) patients in the SGLT-2is group vs. 5 (4.2%) in the TZD group. Non-fatal MIs occurred in 3 (2.5%) vs. 7 (5.8%), and strokes in 1 (0.8%) vs. 4 (3.3%) in the respective groups. Hospitalization for HF was also markedly lower in the SGLT-2is group (3.3%) compared to the TZD group (8.3%) (Table 4).

**Table 4:** Cardiovascular events during follow-up *p<0.05 is considered statistically significant. Events are reported as frequency and percentage, n (%), within each treatment group (SGLT-2is, n=120; TZDs, n=120). Group comparisons were conducted using Pearson’s chi-square (χ²) test; Fisher’s exact test was used when expected cell counts were <5. TZD, thiazolidinedione; SGLT-2is, sodium-glucose co-transporter 2 inhibitors; MACE, major adverse cardiovascular events; MI, myocardial infarction; HF, heart failure

Event	SGLT-2is (n=120)	TZDs (n=120)	χ² value	p-value
Cardiovascular death	2 (1.7%)	5 (4.2%)	1.25	0.263
Non-fatal MI	3 (2.5%)	7 (5.8%)	1.64	0.200
Non-fatal stroke	1 (0.8%)	4 (3.3%)	1.87	0.171
HF hospitalization	4 (3.3%)	10 (8.3%)	3.00	0.083
Total MACE	10 (8.3%)	26 (21.7%)	6.87	0.009*

The Kaplan-Meier survival curves indicated a significantly higher MACE-free survival in the SGLT-2i group compared to the TZD group over the 12-month follow-up period. At the end of the study, the cumulative MACE-free survival probability was 91.7% for the SGLT-2is group and 78.3% for the TZD group. The log-rank test showed a statistically significant difference between the two survival curves (p=0.009), confirming that patients on SGLT-2is experienced significantly fewer cardiovascular events compared to those on TZDs (Table 5).

**Table 5:** Cox regression analysis for MACE TZD, thiazolidinedione; SGLT-2is, sodium-glucose co-transporter 2 inhibitors; MACE, major adverse cardiovascular events; HR, hazard ratio; SBP, systolic blood pressure, LDL, low-density lipoprotein

Covariate	HR	95% CI	p-value
Group (TZD vs. SGLT-2is)	2.18	1.08-4.38	0.029
Age (years)	1.02	0.97-1.06	0.375
Sex (male)	1.15	0.62-2.11	0.659
HbA1c (%)	1.42	1.03-1.96	0.031
LDL (mg/dL)	1.01	0.99-1.02	0.137
SBP (mmHg)	1.03	1.00-1.07	0.048

## Discussion

This prospective, comparative study evaluated the effectiveness of SGLT-2is versus TZDs in reducing ACVEs in patients with T2DM over a 12-month period. The results demonstrate a favorable cardiovascular and metabolic profile for SGLT-2is compared to TZDs, in alignment with several findings from previously published trials and meta-analyses.

At baseline, although both groups were broadly similar in terms of age, gender distribution, and duration of T2DM, the TZD group demonstrated a slightly higher mean BMI (30.87 vs. 29.68 kg/m²) and a greater proportion of smokers (36% vs. 19%). These imbalances are important potential confounders that may have partly contributed to the less favorable cardiovascular outcomes in the TZD group. Smoking is a well-established independent risk factor for atherosclerosis and CVD [9], and its higher prevalence could have contributed to increased cardiovascular events in the TZD group.

After 12 months, the SGLT-2i group exhibited superior improvements in blood pressure and BMI compared to the TZD group. SBP decreased more significantly in the SGLT-2is group (130.5±9.1 mmHg vs. 134.7±10.2 mmHg), which is consistent with previous cardiovascular outcome trials such as EMPA-REG OUTCOME and CANVAS, where SGLT-2is were shown to exert modest but clinically meaningful reductions in SBP [10]. The reduction in blood pressure is likely multifactorial, attributed to osmotic diuresis, natriuresis, and weight loss [11].

The TZD group, in contrast, experienced less pronounced reductions in SBP and BMI. TZDs have been associated with fluid retention and weight gain due to their PPARγ-mediated effects on sodium reabsorption and adipocyte differentiation [12]. In our study, this may explain the attenuated improvement in BMI and SBP observed with TZD therapy.

In terms of glycemic control, SGLT-2is showed a statistically superior reduction in HbA1c and FBG levels compared to TZDs (HbA1c: 7.1±0.7% vs. 7.4±0.8%; FBG: 115.6 vs. 123.8 mg/dL). While both drug classes have established efficacy in improving glycemia, the additional mechanisms of SGLT-2is, such as insulin-independent urinary glucose excretion, likely contribute to better control in this population [13].

Lipid profiles further supported the cardiometabolic advantage of SGLT-2is. Patients in the SGLT-2is group exhibited lower levels of total cholesterol and LDL cholesterol and higher HDL cholesterol, compared to the TZD group. Although pioglitazone has been reported to increase HDL and reduce triglycerides, its effects on LDL are variable [14]. The modest improvement in HDL seen in both groups aligns with prior studies, but the favorable LDL and total cholesterol outcomes in the SGLT-2is group mirror findings from real-world data and RCTs showing improvements in lipid parameters, possibly linked to reduced visceral adiposity and insulin resistance [15].

Both groups maintained stable renal function throughout the study. The mean eGFR remained above 80 mL/min/1.73m², with no significant difference between groups. However, SGLT-2is have been shown to confer long-term renal protection by reducing intraglomerular pressure and albuminuria [16]. Although our study did not assess long-term renal outcomes or albuminuria, the maintenance of renal function over 12 months in the SGLT-2is group is reassuring and consistent with findings from the CREDENCE and DAPA-CKD trials [17].

The incidence of MACE, comprising cardiovascular death, non-fatal MI, non-fatal stroke, and HF hospitalization, was significantly lower in the SGLT-2is group (8.3%) compared to the TZD group (21.7%). This finding is consistent with previous cardiovascular outcome trials, which reported significant reductions in cardiovascular death and HF hospitalization with SGLT-2is [18,19].

Cardiovascular death occurred less frequently in the SGLT-2is group (1.7% vs. 4.2%), and this aligns with the EMPA-REG trial, where empagliflozin demonstrated a 38% relative risk reduction in cardiovascular mortality [20]. Non-fatal MI and stroke were also lower in the SGLT-2is group, albeit not reaching statistical significance due to the limited sample size. However, in the Korean real-world study by Lee et al. [21], the rates of stroke and MI were similar between SGLT-2is and TZD users, though SGLT-2i users had significantly fewer HF hospitalizations (HR: 0.645, 95% CI: 0.466-0.893).

Our findings corroborate the reduction in HF hospitalization (3.3% vs. 8.3%) in favor of SGLT-2is. The hemodynamic and diuretic effects of these drugs likely contribute to improved cardiac loading conditions, thereby reducing HF risk [22]. On the other hand, TZDs, particularly pioglitazone, are known to increase fluid retention and precipitate HF in susceptible individuals, which supports the higher HF hospitalization rates in our TZD group [23].

Interestingly, the results of our study slightly diverge from the Korean study in terms of stroke outcomes, as we observed fewer stroke events in the SGLT-2is group. This discrepancy might be due to differences in population characteristics, follow-up duration, or the proportion of patients with prior cerebrovascular events. Notably, the protective effect of pioglitazone on stroke has been most pronounced in secondary prevention settings, such as the PROactive and IRIS trials [24], while in primary prevention cohorts, its benefit is less clear. Thus, while SGLT-2is generally appear more favorable, TZDs may still hold therapeutic value in carefully selected populations, particularly for secondary stroke prevention.

The Kaplan-Meier survival analysis clearly demonstrated that patients treated with SGLT-2is had significantly better cardiovascular outcomes than those treated with TZDs, consistent with findings from large-scale trials like EMPA-REG OUTCOME and CANVAS. The survival benefit observed in the SGLT-2is group is in line with the known mechanisms of SGLT-2is, such as volume reduction, blood pressure lowering, and improved myocardial energy efficiency, which collectively reduce cardiovascular strain.

The Cox proportional hazards model further validated this association. After adjusting for confounding variables (age, sex, HbA1c, LDL cholesterol, and systolic blood pressure), TZD use remained significantly associated with a 2.18-fold higher risk of MACE compared to SGLT-2is (p=0.029). This suggests that even after accounting for baseline cardiovascular risk factors, the difference in drug class independently influences cardiovascular event rates.

Among the covariates, HbA1c was also a significant predictor of cardiovascular events (HR=1.42, p=0.031), underscoring the role of glycemic control in macrovascular complications. Additionally, higher systolic blood pressure was associated with increased cardiovascular risk (HR=1.03, p=0.048), consistent with previous epidemiologic data. Interestingly, age, sex, and LDL cholesterol were not significant predictors [21,25] in this model, possibly due to relatively uniform baseline characteristics and sample size limitations.

A major strength of this study lies in its prospective design and real-world applicability within a South Asian cohort, where data on comparative antidiabetic cardiovascular outcomes remain scarce. However, the relatively short duration of follow-up, limited sample size, and baseline imbalances in smoking and BMI should be acknowledged as important limitations, as these may have influenced the observed cardiovascular outcomes. Moreover, as the study was conducted in a South Asian population, the generalizability of these findings to other ethnic groups may be limited.

## Conclusions

This prospective study demonstrates that in this South Asian cohort, SGLT-2is were associated with greater improvements in glycemic control, reductions in blood pressure and BMI, and lower risk of MACE (including MI, stroke, and cardiovascular death) compared to TZDs. While these findings are encouraging, they should be interpreted with caution, given the non-randomized single-center design and potential baseline imbalances. Confirmation in larger, multi-center studies with longer follow-up and comprehensive safety evaluations is warranted.

## References

[1] Ma CX, Ma XN, Guan CH, Li YD, Mauricio D, Fu SB (2022). Cardiovascular disease in type 2 diabetes mellitus: progress toward personalized management. Cardiovasc Diabetol.

[2] Rashid SMA, Hossain SM (2022). Stroke and coronary heart diseases, global and Asian trend and risk factors-a perspective. Medicine Today.

[3] Sohn M, Dietrich JW, Nauck MA, Lim S (2023). Characteristics predicting the efficacy of SGLT-2 inhibitors versus GLP-1 receptor agonists on major adverse cardiovascular events in type 2 diabetes mellitus: a meta-analysis study. Cardiovasc Diabetol.

[4] Khan U, Amin AM, Mohamed Taha A (2025). The effect of sodium-glucose co-transporter 2 inhibitors on clinical outcomes after acute myocardial infarction: a systematic review and meta-analysis of randomized controlled trials. Future Cardiol.

[5] Tsai WH, Chuang SM, Liu SC (2021). Effects of SGLT2 inhibitors on stroke and its subtypes in patients with type 2 diabetes: a systematic review and meta-analysis. Sci Rep.

[6] Kounatidis D, Vallianou NG, Rebelos E (2025). The many facets of PPAR-γ agonism in obesity and associated comorbidities: benefits, risks, challenges, and future directions. Curr Obes Rep.

[7] Spence JD, Viscoli C, Kernan WN (2022). Efficacy of lower doses of pioglitazone after stroke or transient ischaemic attack in patients with insulin resistance. Diabetes Obes Metab.

[8] Ohira T, Shahar E, Chambless LE, Rosamond WD, Mosley TH Jr, Folsom AR (2006). Risk factors for ischemic stroke subtypes: the Atherosclerosis Risk in Communities study. Stroke.

[9] Fagerberg B, Barregard L (2021). Review of cadmium exposure and smoking-independent effects on atherosclerotic cardiovascular disease in the general population. J Intern Med.

[10] Yu J, Zhou Z, Mahaffey KW (2021). An exploration of the heterogeneity in effects of SGLT2 inhibition on cardiovascular and all-cause mortality in the EMPA-REG OUTCOME, CANVAS Program, DECLARE-TIMI 58, and CREDENCE trials. Int J Cardiol.

[11] Katsimardou A, Theofilis P, Vordoni A, Doumas M, Kalaitzidis RG (2024). The effects of SGLT2 inhibitors on blood pressure and other cardiometabolic risk factors. Int J Mol Sci.

[12] Singh G, Kumar R, Desna DS, Chaudhary M, Kaur C, Khurrana N (2024). Thiazolidinedione as a promising medicinal scaffold for the treatment of type 2 diabetes. Curr Diabetes Rev.

[13] Yabiku K (2021). Efficacy of sodium-glucose cotransporter 2 inhibitors in patients with concurrent type 2 diabetes mellitus and non-alcoholic steatohepatitis: a review of the evidence. Front Endocrinol (Lausanne).

[14] Premji R, Nylen ES, Naser N, Gandhi S, Burman KD, Sen S (2022). Lipid profile changes associated with SGLT-2 inhibitors and GLP-1 agonists in diabetes and metabolic syndrome. Metab Syndr Relat Disord.

[15] Bechmann LE, Emanuelsson F, Nordestgaard BG, Benn M (2024). SGLT2-inhibition increases total, LDL, and HDL cholesterol and lowers triglycerides: meta-analyses of 60 randomized trials, overall and by dose, ethnicity, and drug type. Atherosclerosis.

[16] Scheen AJ, Delanaye P (2022). Understanding the protective effects of SGLT2 inhibitors in type 2 diabetes patients with chronic kidney disease. Expert Rev Endocrinol Metab.

[17] Heerspink HJ, Oshima M, Zhang H (2022). Canagliflozin and kidney-related adverse events in type 2 diabetes and CKD: findings from the randomized CREDENCE trial. Am J Kidney Dis.

[18] Talha KM, Anker SD, Butler J (2023). SGLT-2 inhibitors in heart failure: a review of current evidence. Int J Heart Fail.

[19] Singh AK, Singh R (2021). Cardiovascular Outcomes with SGLT-2 inhibitors in patients with heart failure with or without type 2 diabetes: a systematic review and meta-analysis of randomized controlled trials. Diabetes Metab Syndr.

[20] Fitchett D, Zinman B, Inzucchi SE (2024). Effect of empagliflozin on total myocardial infarction events by type and additional coronary outcomes: insights from the randomized EMPA-REG OUTCOME trial. Cardiovasc Diabetol.

[21] Lee SE, Nam H, Choi HS, Kim H, Kyoung DS, Kim KA (2022). Comparative effects of sodium-glucose cotransporter 2 inhibitor and thiazolidinedione treatment on risk of stroke among patients with type 2 diabetes mellitus. Diabetes Metab J.

[22] Scheen AJ (2022). Counteracting heart failure with diabetes drugs: a review into the pharmacokinetic and pharmacodynamic properties. Expert Opin Drug Metab Toxicol.

[23] Nesti L, Tricò D, Mengozzi A, Natali A (2021). Rethinking pioglitazone as a cardioprotective agent: a new perspective on an overlooked drug. Cardiovasc Diabetol.

[24] Azhari H, Hewitt J, Smith A, O'Neill M, Quinn T, Dawson J (2024). Pioglitazone and barriers to effective post-stroke comorbidity management in stroke survivors with diabetes. Neurosciences (Riyadh).

[25] Rhee JJ, Han J, Montez-Rath ME, Chertow GM (2024). Comparative effectiveness of sodium-glucose cotransporter-2 inhibitors versus glucagon-like peptide-1 receptor agonists in patients with type 2 diabetes and mild/moderate chronic kidney disease. Diabetes Obes Metab.

